# Monitoring for 5-aminosalicylate nephrotoxicity in adults with inflammatory bowel disease: prognostic model development and validation using data from the Clinical Practice Research Datalink

**DOI:** 10.1136/bmjgast-2024-001627

**Published:** 2025-01-25

**Authors:** Abhishek Abhishek, Georgina Nakafero, Tim Card, Maarten W Taal, Matthew J Grainge, Guruprasad P Aithal, Christian D Mallen, Matthew D Stevenson, Richard D Riley

**Affiliations:** 1 Academic Rheumatology, University of Nottingham, Nottingham, East Midlands, UK; 2 Nottingham NIHR BRC, Nottingham, UK; 3 Lifespan and Population Health, School of Medicine, University of Nottingham, Nottingham, UK; 4 Centre for Kidney Research and Innovation, Translational Medical Sciences, University of Nottingham, Nottingham, UK; 5 Nottingham Digestive Diseases Centre, Translational Medical Sciences, School of Medicine, University of Nottingham, Nottingham, UK; 6 Primary Care Centre Versus Arthritis, Keele University, Keele, UK; 7 School of Health and Related Research, University of Sheffield, Sheffield, UK; 8 Institute of Applied Health Research, College of Medical and Dental Sciences, University of Birmingham, Birmingham, UK; 9 NIHR, Birmingham Biomedical Research Centre, Birmingham, UK

**Keywords:** 5-AMINOSALICYLIC ACID (5-ASA), CROHN'S COLITIS, ULCERATIVE COLITIS

## Abstract

**Objective:**

To develop and validate a prognostic model for risk-stratified monitoring of 5-aminosalicylate nephrotoxicity.

**Methods:**

This UK retrospective cohort study used data from the Clinical Practice Research Datalink Aurum and Gold for model development and validation respectively. It included adults newly diagnosed with inflammatory bowel disease and established on 5-aminosalicylic acid (5-ASA) treatment between 1 January 2007 and 31 December 2019. Drug discontinuation associated with 5-ASA nephrotoxicity defined as a prescription gap of ≥90 days with decline in kidney function was the outcome. Patients prescribed 5-ASAs for ≥6 months were followed-up for up to 5 years. Penalised Cox regression was used to develop the risk equation with bootstrapping for internal validation and optimism adjustment. Model performance was assessed in terms of calibration and discrimination.

**Results:**

13 728 and 7318 participants who contributed 40 378 and 20 679 person-years follow-up formed the development and validation cohorts with 170 (1.2%) and 98 (1.3%) outcome events respectively. Nine predictors were included in the final model, including chronic kidney disease stage 3 and hazardous alcohol use as strong predictors. Age and Body Mass Index were weak predictors. The optimism-adjusted calibration slope, C and D statistics in the development and validation data were 0.90, 0.64 and 0.98, and 1.01, 0.66 and 0.94 respectively.

**Conclusion:**

This prognostic model used information from routine clinical care and performed well in an independent validation cohort. It can be used to risk-stratify blood test monitoring during established 5-ASA treatment. A key limitation is that the decline in kidney function could have been due to factors other than 5-ASA nephrotoxicity.

**WHAT IS ALREADY KNOWN ON THIS TOPIC**

**WHAT THIS STUDY ADDS**

- This study developed and externally validated a prognostic model to estimate the risk of nephrotoxicity due to 5-aminosalicylates during established treatment using a large dataset from the UK that originated during routine clinical care.
- Most patients were at low risk of 5-aminosalicylate nephrotoxicity and could continue with annual monitoring blood tests while others at high risk may require frequent monitoring.
- This prognostic model can be used to help inform decisions on the interval between monitoring blood tests, thereby, improving patient safety.

**HOW THIS STUDY MIGHT AFFECT RESEARCH, PRACTICE OR POLICY**

- These findings should be considered by guideline writing groups to provide risk-based recommendations for the frequency of monitoring of kidney function in people with inflammatory bowel disease receiving treatment with 5-aminosalicylic acid.

## Introduction

5-aminosalicylates (5-ASA) are the mainstay of treatment for mild to moderate ulcerative colitis and are often combined with biologics in the treatment of severe disease.^1^,^3^ Although effective, they can cause potentially serious adverse effects such as acute interstitial nephritis.^4^,^9^ Clinically significant interstitial nephritis due to 5-ASA drugs is estimated to occur in 1 in 500 patients and may reverse on drug discontinuation.^7 10^ This makes screening for renal toxicity important. Although 5-ASA-induced interstitial nephritis is more common in the first year of treatment,^8 9^ it often occurs later in the treatment course with a median time to onset of between 2.3 and 3 years reported.^6 7^

Patients established on 5-ASA treatment are recommended to undergo periodic monitoring of kidney function. There is currently no consensus on the frequency of testing due to lack of high-quality data. The British Society of Gastroenterology (BSG) guideline and the British National Formulary (BNF) recommend monitoring of kidney function at annual intervals in those established on treatment, with consideration of increased frequency in those with impaired renal function.^1 11^ Others advise either 6 monthly, or annual or ‘periodic monitoring at the clinician’s discretion’.^1^,^312^ The recommendation to monitor is often not followed, potentially, due to the rarity of 5-ASA nephrotoxicity. For example, only a fifth of patients underwent monitoring blood tests in a large UK study.^13^

Current recommendations generally adopt a one-size-fits-all approach to monitoring for 5-ASA nephrotoxicity. This is potentially a wasteful use of resources while disadvantaging others at high risk. It would be beneficial to predict nephrotoxicity during established 5-ASA treatment to allow risk-stratified monitoring. In this study, we developed and validated a prognostic model for 5-ASA nephrotoxicity during established treatment to aid risk-stratified long-term monitoring strategies.

## Methods

### Data source

Clinical Practice Research Datalink (CPRD).^14 15^ CPRD is an anonymised longitudinal database of electronic health records collected during routine clinical care in the National Health Service. With almost universal coverage of UK residents, participants that contributed data to the CPRD are representative of the UK population.^14^ CPRD Aurum and CPRD Gold are two separate CPRD data sets which use different practice management software and complement each other in terms of coverage of general practices. CPRD Aurum only includes practices from England while GOLD contains practices from other parts of the UK. For the purposes of this study, CPRD Aurum and Gold were used for model development and validation, respectively. Some general practices which have contributed data to both databases at different time were only included in the model development cohort.

### Study design

Retrospective cohort study.

### Study period

1 January 2007 to 31 December 2019.

### Study population

Participants aged ≥18 years with a new diagnosis of inflammatory bowel disease (IBD) and newly prescribed 5-ASA by their general practitioner (GP) for at least 180 days were eligible. Patients with chronic kidney disease (CKD) stage ≥4 prior to first 5-ASA prescription were excluded (online supplemental figures S1 and S2).

### Follow-up

Patients were followed-up from 180 days after their first GP prescription until the earliest of outcome, death, transfer out of practice, 90-day prescription gap, last data collection from practice, 31 December 2019 or 5 years from the start of follow-up.

Follow-up began 180 days after first GP prescription as our purpose was to predict the risk of 5-ASA nephrotoxicity during established treatment, a period in which there is greatest need to risk-stratify monitoring as the rate of nephrotoxicity is lower than early in the treatment course.

In the UK, 5-ASA drugs are commenced in hospital clinics. Hospital specialists prescribe and monitor these drugs till a stable well-tolerated dose is reached, and the monitoring blood tests are satisfactory. This takes approximately 6 months. After this, the responsibility for prescribing and monitoring is handed over to GPs. Consequently, we started follow-up from 180 days after the first GP prescription to only include patients treated with 5-ASA drugs for approximately 1 year.

### Outcome

5-ASA nephrotoxicity-associated drug discontinuation was the outcome of interest. This was defined as a prescription gap of ≥90 days with decline in kidney function, defined as either progression of CKD based on medical codes recorded by the GP, or >26 µmol/L increase in creatinine concentration, the threshold for consideration of acute kidney injury (AKI)^16^ within ±60 days of the last prescription.

To assess the positive predictive value of the outcome, a random sample of 5-ASA discontinuations with abnormal blood test results was drawn. Data for all diagnostic codes entered during primary-care consultations within ±60 days of the abnormal blood test result were extracted. A.A. screened the list to identify outcomes that could potentially be explained by an alternative condition or its treatment.

### Candidate predictors

Candidate predictors were selected based on clinical expertise and knowledge of the published literature (table 1). Age, sex Body Mass Index (BMI), alcohol intake and diabetes were included as they are associated with worsening renal function.^17^ Additionally, diabetes has been associated with 5-ASA nephrotoxicity.^18^ CKD stage 3 was included as it reduces 5-ASA clearance and has been associated with 5-ASA nephrotoxicity.^11 19^ Prescriptions of ACE inhibitors, aspirin and non-steroidal anti-inflammatory drugs (including COX-2 inhibitors) were considered as their use is associated with 5-ASA nephrotoxicity as per the British National Formulary^11^ and a previous study.^18^ 5-ASA dose was not included in the model as 5-ASA-induced nephrotoxicity is reported to be idiosyncratic and not dose-related.^9^

**Table 1:** Distribution of candidate predictors in development and validation cohorts

Predictor*	Development cohort(CPRD Aurum)n=13 728	Validation cohort(CPRD Gold)n=7318
Age, mean (SD) year	46 (18)	47 (18)
Male sex	7075 (51.5)	3772 (51.5)
Body Mass Index		
<18.5 kg/m^2^	430 (3.1)	229 (3.1)
18.5–24.9 kg/m^2^	4914 (35.8)	2480 (33.9)
25.0–29.9 kg/m^2^	3934 (28.7)	2172 (29.7)
≥30 kg/m^2^	2563 (18.7)	1436 (19.6)
Missing	1887 (13.8)	1001 (13.7)
Alcohol use		
Non-user	298 (16.7)	910 (12.4)
Low (1–14 units/week)	5688 (41.4)	3707 (50.7)
Moderate (15–21 units/week)	819 (6.0)	421 (5.8)
Hazardous (>21 units/week)	986 (7.2)	400 (5.5)
Ex-user	1041 (7.6)	384 (5.3)
Missing	2896 (21.1)	1496 (20.4)
Comorbidities		
Diabetes	1043 (7.6)	554 (7.6)
Chronic kidney disease stage 3	797 (5.8)	399 (5.4)
Other drugs		
ACE inhibitors	1279 (9.3)	732 (10.0)
Aspirin	991 (7.2)	621 (8.5)
NSAIDs including COX-2 inhibitors	635 (4.6)	308 (4.2)

* Values are numbers (percentage) unless stated otherwise.

CPRD, Clinical Practice Research DatalinkNSAIDsnon-steroidal anti-inflammatory drugs

### Sample size

The reported incidence of interstitial nephritis due to 5-ASAs ranged between 1.2 and 1.7 per 1000 person-years.^4 20^ Using the formulae of Riley *et al*,^21^ the minimum sample size required for new model development was 1049 participants (43 events) based on a maximum of 15 parameters, and assumed Cox-Snell R^2^ value of 0.12, estimated event rate of 0.005/person-year and a mean follow-up period of 3 years to minimise model overfitting and ensure precise estimation of overall risk at 5 years. Our sample size within CPRD far exceeded this (online supplemental figures S1 and S2).

### Statistical analysis

Multiple imputations handled missing data on BMI and alcohol intake using chained equations.^22^ We carried out 10 imputations in the development dataset and 5 imputations in the validation dataset—a pragmatic approach considering the large size of CPRD. The imputation model included all candidate predictors, baseline Nelson-Aalen cumulative hazard function and outcome variable.

*Model development*: All candidate predictors and parameters were included in the Cox model (ie, no variable selection was used) and coefficients of each parameter were estimated and combined using Rubin’s rule across the imputed datasets. We compared nested models containing each continuous predictor in turn with that containing the best-fitting first-order polynomial. It was found that for both age and BMI, fractional terms did not improve model fit (p value=0.217 and 0.359 respectively); hence, these variables were not transformed. Patients who died were censored at their death; thus, the model gives estimated 5-year risks conditional on being alive at 5 years which is highly likely for most patients, see Discussion.

The risk equation for predicting an individual’s risk of 5-ASA discontinuation with nephrotoxicity-associated drug discontinuation by 5 years follow-up was formulated in the development data. The baseline survival function at t=5 years, a non-parametric estimate of survival function when all predictor values are set to zero, which is equivalent to the Kaplan-Meier product-limit estimate, was estimated along with the estimated Cox regression coefficients (β). This led to the equation for the predicted absolute risk over time.^23^

Predicted risk=1 – S_0_(t_=5_) ^exp(Xβ)^, where S_0_(t_=5_) is the baseline survival function at 5 years of follow-up and βX is the linear predictor, β_1_x_1_+ β_2_x_2_+ … + β_p_x_p_, with the individual’s predictor values denoted by X.

### Model internal validation and shrinkage

Apparent performance (in the development dataset) of the model was assessed in terms of calibration and discrimination. Calibration examines the agreement between predicted and observed risks and can be summarised by a calibration plot (with smooth calibration curve) and calibration slope (ideal value of 1). Discrimination was measured using the Harrell’s C-statistic, a measure of the model’s predictive accuracy, and Royston D statistic, interpreted as a log HR comparing the outcome rate of two groups defined by the median of the model’s linear predictor.^24 25^ Overall fit was quantified by R^2^_D_, a measure of variation explained by the model.

Internal validation was performed using bootstrapping with replacement, with 500 bootstrap samples used to estimate and adjust performance estimates for optimism due to potential overfitting. The full model was fitted in each bootstrap sample and then its performance quantified in the bootstrap sample (apparent performance) and the original sample (test model performance), and the optimism calculated (difference in test performance and apparent performance) for each measure of calibration and discrimination performance. A uniform shrinkage factor was estimated as the average of optimism-adjusted calibration slopes from the bootstrap sample. This process was repeated for all 10 imputed datasets, and the final uniform shrinkage calculated by averaging across the estimated shrinkage estimates from each imputation. Optimism-adjusted estimates of performance for the original model were then calculated, as the original apparent performance minus the average optimism.

To account for overfitting, the original β coefficients were multiplied by the final uniform shrinkage factor and the baseline hazard re-estimated with the shrunken β coefficients as an offset; this ensured that overall calibration was maintained and produced the final model.

### Model external validation

The final developed model equation was applied to individuals in the validation dataset, and calibration and discrimination were examined.^24 25^ Calibration of 5-year risks was examined by plotting agreement between estimated risk from the model and ‘observed’ outcome risks. Pseudo-observations were used to construct smooth calibration curves across all individuals via a running non-parametric smoother. We also examined predicted and observed risks within 10 equally sized groups defined by tenths of predicted risk. Separate calibration plots were considered for each imputation. Stata-MP version 17 was used for statistical analyses. This study was reported in line with the TRIPOD guidelines (see TRIPOD checklist [online supplemental]).^26^

## Results

### Participants

Data for 13 728 and 7318 participants that contributed 40 378 and 20 679 person-years follow-up were included in the development and validation cohorts, respectively (online supplemental figures S1 and S2). In the development cohort, 9096 (66%) had ulcerative colitis, 2769 (20%) had Crohn’s disease, and 1863 (14%) had IBD without any specific coding for subtype. In the validation cohort, 4449 (61%) had ulcerative colitis, 1539 (21%) had Crohn’s disease, and 1330 (18%) had IBD without any specific coding for subtype. Over 50% were recorded as male and had similar lifestyle factors, comorbidities and drug treatments (table 1). Nine candidate predictors (12 predictor parameters) were included in the model (table 2). We were unable to include lithium, ciclosporin, voclosporin and methotrexate, drugs associated with 5-ASA nephrotoxicity,^11 18^ as potential predictors in the prognostic model because there were a small number of patients prescribed these drugs in the development cohort and none of them had the outcome of interest.

**Table 2:** Cox model HRs and β-coefficients before penalisation (shrinkage)

Predictors	Adjusted HR(95% CI)	Coefficients	Shrunken coefficients*
Age, year	1.01 (1.00, 1.02)	0.0096984	0.00872856
Female sex	0.73 (0.52, 1.01)	−0.3190717	−0.28716453
Body Mass Index (kg/m^2^)	1.03 (1.00, 1.05)	0.0259328	0.02333952
Alcohol use			
Non-user	Reference	–	
Low (1–14 units/week)	1.38 (0.84, 2.56)	0.320509	0.2884581
Moderate (15–21 units/week)	1.10 (0.46, 2.61)	0.0962384	0.08661456
Hazardous (>21 units/week)	2.26 (1.17, 4.36)	0.8169186	0.73522674
Ex-user	2.01 (1.08, 3.77)	0.7002583	0.63023247
Comorbidities			
Diabetes	1.26 (0.79, 1.99)	0.2276251	0.20486259
Chronic kidney disease stage 3	2.98 (1.96, 4.53)	1.09148	0.982332
Other drugs			
ACE inhibitors	1.34 (0.88, 2.03)	0.2938861	0.26449749
Aspirin	1.30 (0.84, 2.03)	0.2653679	0.23883111
NSAIDs	1.14 (0.63, 2.06)	0.1298207	0.11683863

* Multiplied by the shrinkage factor (0.9).

NSAIDsnon-steroidal anti-inflammatory drugs

### Model development

In the development dataset, there were 25 (0.18%) deaths. 170 outcomes occurred in n=13 728 patients (1.2%) during follow-up at a rate (95% CI) of 4.21 (3.62–4.89) per 1000 person-years. These outcomes occurred at a similar rate throughout the follow-up period (online supplemental figure S3). The median (IQR) time to occurrence of outcome was 1.66 (0.80–3.04) years. Outcome validation exercise revealed that 13.7% outcomes (n=10/73) could potentially be explained by another contemporaneous illness (vomiting, diarrhoea, membranoproliferative nephritis unspecified, gastroenteritis, acute myocardial infarction, congestive cardiac failure, urology referral and admission to medical emergency ward (with unspecified reason) with a positive predictive value of 86%. Pre-existing CKD stage 3, hazardous alcohol use and ex-alcohol use were strong predictors of drug discontinuation with adjusted HR (95% CI) before shrinkage of 2.98 (1.96, 4.53), 2.26 (1.17, 4.36) and 2.01 (1.08, 3.77) respectively (table 2). Before shrinkage, the calibration slope in the development data was 1.00 (95% CI 0.81 to 1.19). From the bootstrap process, the estimated uniform shrinkage factor (optimism-adjusted calibration slope) was 0.90 and this was used to shrink predictor coefficients for optimism, to form the final model shown in box 1, including, after re-estimation, the cumulative baseline survival function (S_0_) of 0.994 at 5 years of follow-up.

Box 1 **Equation to predict the risk of 5-ASA discontinuation after 6 months of primary care prescription and within the next 5 years**

Risk score = 1 – 0.994 ^exp(0.90βX)^, where βX= 0.0096984 * age in years at first primary-care prescription − 0.3190717 * female sex + 0.0259328 * Body Mass Index + 0.320509 * low alcohol intake + 0.0962384 * moderate alcohol intake + 0.8169186 * hazardous alcohol intake + 0.7002583 * ex-alcohol intake + 0.2276251 * diabetes + 1.09148 * chronic kidney disease stage 3 + 0.2938861 * ACE inhibitors + 0.2653679 * aspirin + 0.1298207 * NSAIDs.

All variables are code 0, and 1 if absent or present respectively, except for BMI and age that are continuous variables. 0.994 is the baseline survival function at 5 years, 0.90 is the shrinkage factor and the other numbers are the estimated regression coefficients for the predictors, which indicate their mutually adjusted relative contribution to the outcome risk.

### Model performance in the development cohort

Calibration plot of the final (ie, after shrinkage) model at 5 years is shown in online supplemental figure S4. As most patients had low risk of outcome, the groups defined by tenths of predicted risk are mostly clustered at the bottom left of the calibration plot (online supplemental figure S5). The smoothed calibration curve at 5 years showed alignment of observed risk to the predicted risk with wide CIs at >0.1 risk probabilities (online supplemental figure S4). The Royston D statistic (95% CI) was 1.13 (0.88, 1.38) corresponding to an HR of 3.10 (2.44–3.97) when comparing the risk groups above and below the median of linear predictor. The optimism-adjusted Royston D statistic was 0.98 corresponding to an HR of 2.66. The optimism-adjusted C-statistic and R^2^_D_ were 0.64 and 0.18 respectively (table 3).

**Table 3:** Model diagnostics

Measure	Apparent performance*	Test performance†	Average optimism‡	Optimism corrected performance§	External validation (CPRD Gold)
Overall calibration slope	1.00(0.81, 1.19)	0.90	0.10	0.90	1.01(0.71, 1.30)
Harrel’s C-statistic	0.67(0.63, 0.72)	0.66	0.02	0.64	0.66 (0.60, 0.72)
R^2^_D_	0.23(0.16, 0.31)	0.21	0.05	0.18	0.17(0.08, 0.27)
Royston D statistic	1.13(0.88, 1.38)	1.05	0.15	0.98	0.94(0.62, 1.26)

* Estimated directly from data that was used to develop the model.

† Determined by executing full model in each bootstrap sample of the development dataset (500 samples with replacement), calculating bootstrap performance, and applying same model in original sample.

‡ Average difference between model performance in bootstrap sample of the development dataset and performance in the development dataset.

§ Obtained by subtracting average optimism from apparent performance.

CPRD, Clinical Practice Research Datalink;

### Model performance in the validation cohort

**Figure 1:**
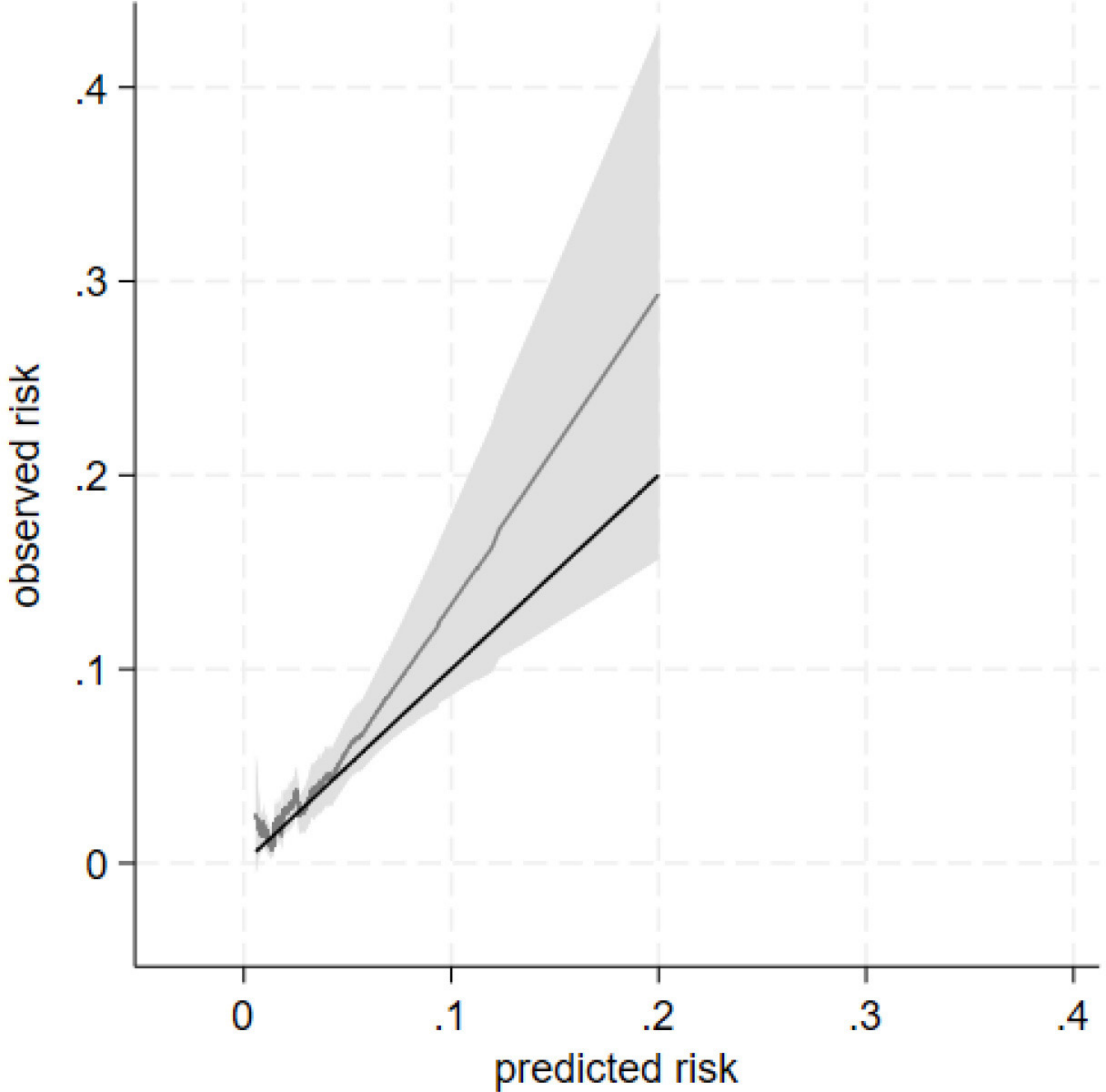
Calibration of a prognostic model for 5-aminosalicylic acid (5-ASA) discontinuation with abnormal monitoring blood test results at 5 years in the validation cohort. Data from a single imputed dataset were used for illustration. Baseline survival function(So) was 0.994 at 5 years. Black line reflects perfect prediction. Grey line shows model prediction with 95% CI in grey shade.

There were 10 (0.14% deaths). 98 outcomes occurred in n=7318 (1.3%) patients at a rate (95% CI) of 4.74 (3.89–5.77) per 1000 person-years in the validation cohort. The calibration slope (95% CI) at the 5-year follow-up period was 1.01 (0.71–1.30). The calibration plot showed reasonable correspondence between observed and predicted risk at 5 years across the tenths of risk with CIs crossing the ideal line of agreement (online supplemental figure S6). As above, most groups clustered at the bottom left of the calibration plot owing to low risk of outcome for most patients (online supplemental figureS7). The smoothed calibration curve showed miscalibration in those at high risk, with under-prediction of risk, although this could be limited by a small number of people at high risk reflected by a wide 95% CI (figure 1). Model performance was also tested at years 1, 2, 3 and 4 and showed a similar pattern (online supplemental figures S11 and S12). Model discrimination in the validation data was broadly like the development data (table 3). The Royston D statistic in the validation data was 0.94 (0.62, 1.26), corresponding to HR (95% CI) 2.56 (1.86–3.53). The R^2^ score was 0.17 (95% CI 0.08, 0.27). Harrell’s C-statistic was 0.66 (95% CI 0.60, 0.72).

### Worked examples

10 anonymised patient profiles, one from the middle of each tenth of predicted risk, were selected from the development cohort (online supplemental table S1). The cumulative probability of outcome over 5 years ranged from 0.9% in the middle of the first group to 1.9% in the middle of the seventh group, and 13.2% in the middle of the 10th group.

## Discussion

### Summary

This study developed and externally validated a prognostic model for 5-ASA discontinuation due to nephrotoxicity defined using a gap in prescription and renal function decline in people with IBD established on 5-ASA treatment that is, after the first 6 months of primary-care prescription. We were able to predict 5-ASA discontinuation due to nephrotoxicity using readily available data. Currently, everyone prescribed 5-ASA is recommended to undergo annual monitoring blood tests for the early detection of 5-ASA nephrotoxicity. As early discontinuation of the drug and treatment with steroids may result in rapid improvement in kidney function, there is a compelling case for monitoring those at a high risk of nephrotoxicity more frequently.^6^ This study also provides data on the incidence of nephrotoxicity due to 5-ASA in a large population established on 5-ASA in primary care. Due to the use of a pragmatic definition, these rates are likely to be an overestimate.

### Strengths and limitations

Strengths of this study include model development and validation using a large real-world and nationally representative dataset that originated during routine clinical care in the UK and therefore, has high generalisability to the UK population. Additionally, the model had good performance characteristics in an independent dataset and predicted clinically relevant nephrotoxicity that is, one that required 5-ASA cessation, providing face validity. The prognostic factors included those that are readily available during routine consultations, making the model easy to use, especially, once incorporated into clinical decision support software.

However, the findings of this study should be interpreted considering several limitations. First, there is some information which we would ideally want but was not available. The date when 5-ASA was first prescribed from hospital was unavailable. We think this is not a serious issue as the model can be applied once the patient has completed 6 months of 5-ASA treatment from primary care after treatment initiation and initial stabilisation in a hospital clinic. Second, we did not have information on the use of biologics as these are hospital prescribed. There is however no reason to believe that concurrent biologic prescription would increase the risk of 5-ASA nephrotoxicity. Third, we also did not have data on the severity of IBD as this is not recorded in CPRD. However, there is no evidence that suggests IBD disease activity is associated with drug-induced side effects.^27 28^ Fourth, we must consider whether the ascertainment of discontinuation being due to nephrotoxicity is reliable. The decline in kidney function could have been due to factors other than 5-ASA-induced nephrotoxicity. Though, a review of potential causes of outcomes in the development cohort revealed that only a minority could potentially be explained by another contemporaneous illness. Our definition of CKD progression relied on coding information and may therefore have been applied variably by different practitioners, and the minimum serum creatinine rise we required to consider a diagnosis of AKI may have included some cases of CKD progression. No kidney biopsy confirmation of 5-ASA nephrotoxicity was available, but this is in keeping with the real-world situation where a decision to stop treatment is usually made before kidney biopsy is considered. Fifth, we did not include genetic factors such as human leucocyte antigen polymorphism that have been associated with 5-ASA nephrotoxicity^7^ as a prognostic factor because such data are not available in the CPRD. Future research should include genetic factors to find out if these would improve the prediction of 5-ASA-associated nephrotoxicity. Sixth, although the external validation dataset was distinct from the model development dataset, it also originated from the UK meaning that generalisability beyond the UK cannot be guaranteed. Therefore, before considering the model in other countries, further validation is required in non-UK data. Finally, we censored individuals who died at their death time rather than modelling death as a competing risk. This means risk predictions at 5 years assume the individual will be alive at that time, which we considered to be clinically more informative for this situation where monitoring decisions are being made.

Our approach as outlined above was inclusive capturing all drug discontinuations possibly due to nephrotoxicity, and hence, our estimates represent a ‘worst-case’ scenario and provide a firm basis for guiding safe practice. Our data show that just over 4 in 1000 IBD patients on 5-ASA will have their medication discontinued possibly due to nephrotoxicity in any year. That this level is rather higher than that of the best previous estimates of the risk of 5-ASA nephrotoxicity may partly be explained by our estimates reflecting the real world and not randomised controlled trials, but equally doubtless implies that in some cases, the drug is discontinued when it is not to blame.^4 5^

### Comparison with existing literature

We observed the rate of drug discontinuation due to apparent nephrotoxicity to be 4.21 and 4.74/1000 person-years in the development and validation cohorts, confirming a low risk of nephrotoxicity associated with 5-ASA treatment. Nevertheless, acute interstitial nephritis is a serious complication which results in end-stage kidney disease in 14.6% of those affected.^6^ Our observed incidence of 5-ASA nephrotoxicity is about threefold lower than the rate reported by Jairath *et al* using data from the THIN database.^18^ However, in their analysis, nephrotoxicity was defined using any diagnostic code for ‘chronic, acute and unspecified renal impairment’ and did not consider drug discontinuation so their definition likely would have included a variety of unrelated kidney diseases.^18^

### Implications for research and/or practice

Our analysis also confirms that, although the risk of nephrotoxicity with 5-ASA treatment is variable across individuals, only a small number of readily available demographic and clinical variables are needed to tailor risk predictions to individuals. This allows the prediction model to be easily used in clinical practice that is, either as an online calculator or embedded in primary care computer clinical decision software solutions. Based on the level of risk we report, current guidelines on monitoring may seem reasonable at a population level. However, we have shown there is a small predictable subset with an appreciably higher risk in whom more frequent monitoring may be appropriate. It is beyond the scope of this study to propose specific risk thresholds at which current clinical practice may be changed. Such recommendations are best made by national and international guideline writing groups considering the views of clinical experts, patients and commissioners. In the UK, the frequency of monitoring blood tests is typically decided according to the recommendations of specialist societies such as the BSG. These findings ought to be considered by them when formulating their guidance.

We recommend that the prognostic model be validated using data from other countries. Further research is needed to examine the impact on patient outcomes of using the model to direct monitoring decisions, for example, using decision analysis methods, cost-effectiveness models and cluster-randomised trials comparing practices that do or do not implement the model.

## Conclusion

We have developed and externally validated a prognostic model for 5-ASA discontinuation due to nephrotoxicity that may be used to individualise blood test monitoring using principles of shared decision making between the patient and the physician. These findings need to be considered by guideline writing groups to provide risk-based recommendations for the frequency of monitoring of kidney function in people with IBD receiving treatment with 5-ASA.

## supplementary material

10.1136/bmjgast-2024-001627 online supplemental file 1

## Data Availability

Data may be obtained from a third party and are not publicly available.
