# Supplementary material for: Monitoring for 5-aminosalicylate nephrotoxicity in adults with inflammatory bowel disease: prognostic model development and validation using data from the Clinical Practice Research Datalink
Source: BMJ Open Gastroenterol. 2025 Jan 25;12(1):e001627. doi: 10.1136/bmjgast-2024-001627 (PMC11784381; doi:10.1136/bmjgast-2024-001627)
Supplement: online supplemental file 1 [file bmjgast-12-1-s001.docx]

**Contents**

[Supplementary Table S1: Individual patient’s characteristics at the midpoint of each decile. 2](#_Toc179276331)

[Figure S1: Study population selection criteria for model development 3](#_Toc179276332)

[Figure S2: Model validation cohort: Study population selection criteria 4](#_Toc179276333)

[Figure S3: Cumulative hazard plot for the occurrence of outcome of interest. 5](#_Toc179276334)

[Figure S4: Calibration plot and smoothed calibration curve of a prognostic model for 5-ASA discontinuation with abnormal monitoring blood-test results at 5-years in the development cohort 6](#_Toc179276335)

[Figure S5: Distribution of predicted risk in the model development cohort at 5 years 7](#_Toc179276336)

[Figure S6: Calibration plot of a prognostic model for 5-ASA discontinuation with abnormal monitoring blood-test results at 5-years in the validation cohort 7](#_Toc179276337)

[Figure S7: Distribution of predicted risk in the model validation cohort at 5 years 9](#_Toc179276338)

[Figure S8: Calibration of a prognostic model for 5-ASA discontinuation with abnormal monitoring blood-test results at 1 year in the validation cohort 10](#_Toc179276339)

[Figure S9: Calibration of a prognostic model for 5-ASA discontinuation with abnormal monitoring blood-test results at 2 years in the validation cohort 11](#_Toc179276340)

[Figure S10: Calibration of a prognostic model for 5-ASA discontinuation with abnormal monitoring blood-test results at 3 years in the validation cohort 12](#_Toc179276341)

[Figure S11: Calibration of a prognostic model for 5-ASA discontinuation with abnormal monitoring blood-test results at 4 years in the validation cohort 13](#_Toc179276342)

## Supplementary Table S1: Individual patient’s characteristics at the midpoint of each decile.

| Decile | Age (Yr.) | Sex | BMI (kg/m^2^) | Alcohol | DM | CKD-3 | ACE-inhibitors | Aspirin | NSAIDs | Cumulative outcome probability (%) |
| --- | --- | --- | --- | --- | --- | --- | --- | --- | --- | --- |
| 1 | 20-25 | F | 30.9 | Non-drinker | 0 | 0 | 0 | 0 | 0 | 0.9 |
| 2 | 15-20 | M | 15.7 | Low | 0 | 0 | 0 | 0 | 0 | 1.1 |
| 3 | 25-30 | M | 18 | Low | 0 | 0 | 0 | 0 | 0 | 1.2 |
| 4 | 20-25 | M | 24.7 | Low | 0 | 0 | 0 | 0 | 0 | 1.4 |
| 5 | 55-60 | F | 29 | Low | 0 | 0 | 0 | 0 | 0 | 1.5 |
| 6 | 40-45 | M | 25.5 | Low | 0 | 0 | 0 | 0 | 0 | 1.7 |
| 7 | 55-60 | M | 24.6 | Low | 0 | 0 | 0 | 0 | 0 | 1.9 |
| 8 | 65-70 | M | 27.8 | Moderate | 1 | 0 | 0 | 0 | 0 | 2.2 |
| 9 | 65-70 | M | 28 | Heavy | 0 | 0 | 0 | 0 | 0 | 3.1 |
| 10 | 80-85 | M | 25.2 | Heavy | 1 | 1 | 0 | 1 | 0 | 13.2 |

BMI: Body Mass Index; ACE-inhibitors: Angiotensin converting enzyme inhibitors; CKD-3: Chronic Kidney Disease stage 3; DM: diabetes mellitus; F: female; M: male, ± 5-year age band. Exact age not shown for anonymity.

## Figure S1: Study population selection criteria for model development

Patients with incident inflammatory bowel disease
 treated with amino salicylates in the study period
n=36,361 (CPRD Aurum 2007-2019)

        Excluded n=22,633

5,859: Registered in general practice surgeries that also contributed data to CPRD Gold.

1,747: < 18 years at diagnosis of inflammatory condition.

5,171: <1 year of registration prior to inflammatory condition diagnosis date.

4,494: Prescribed study drug before inflammatory diagnosis date -90 days.

419: Diagnosed with either chronic liver disease or haematological malignancies or conditions or eGFR <15ml/min or CKD stage 4 or 5 prior to the start of follow-up.

4,112: Discontinued study drug prior to the start of follow-up.

831: Transferred out of GP surgery, died or had outcome prior to start of follow-up.

Included in the development cohort: n=13,728

Aminosalicylates were balsalazide, mesalazine and olsalazine, CKD: chronic kidney disease, GP: General practice, CPRD: Clinical Practice Research Datalink.

## Figure S2: Model validation cohort: Study population selection criteria

Patients with incident inflammatory bowel disease
 treated with amino salicylates in the study period
n=16,117

(CPRD GOLD 2007-2019)

        Excluded n=8,799

921: < 18 years at diagnosis of inflammatory condition.

1,866: <1 year of registration prior to AIRD diagnosis date.
 
3,176: Prescribed study drug before inflammatory condition diagnosis date -90 days.

229: Diagnosed with either chronic liver disease or haematological malignancies or conditions or eGFR <15ml/min or CKD stage 4 or 5 prior to start of follow-up

1,998: Discontinued study drug prior to start of follow-up.

609: Transferred out of GP surgery, died or had outcome prior to start of follow-up.

Included in the validation cohort: n=7,318

Aminosalicylates were balsalazide, mesalazine and Olsalazine, CKD: chronic kidney disease, GP: General practice, CPRD: Clinical Practice Research Datalink.

## Figure S3: Cumulative hazard plot for the occurrence of outcome of interest.


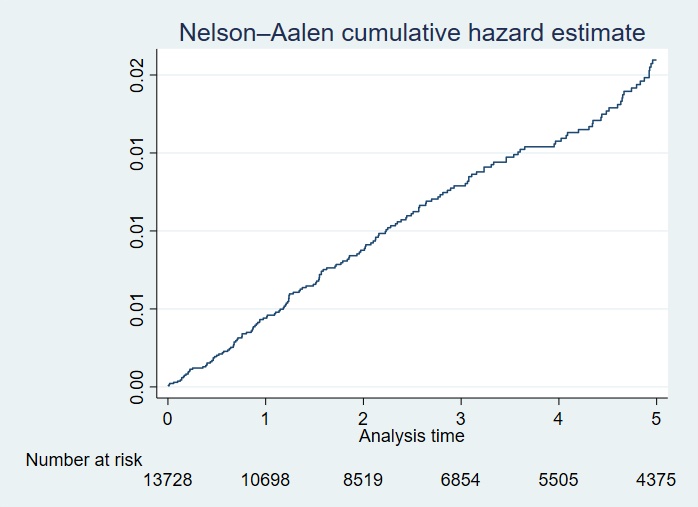


## Figure S4: Calibration plot and smoothed calibration curve of a prognostic model for 5-ASA discontinuation with abnormal monitoring blood-test results at 5-years in the development cohort

4a. Calibration plot 4b. Smoothed calibration curve


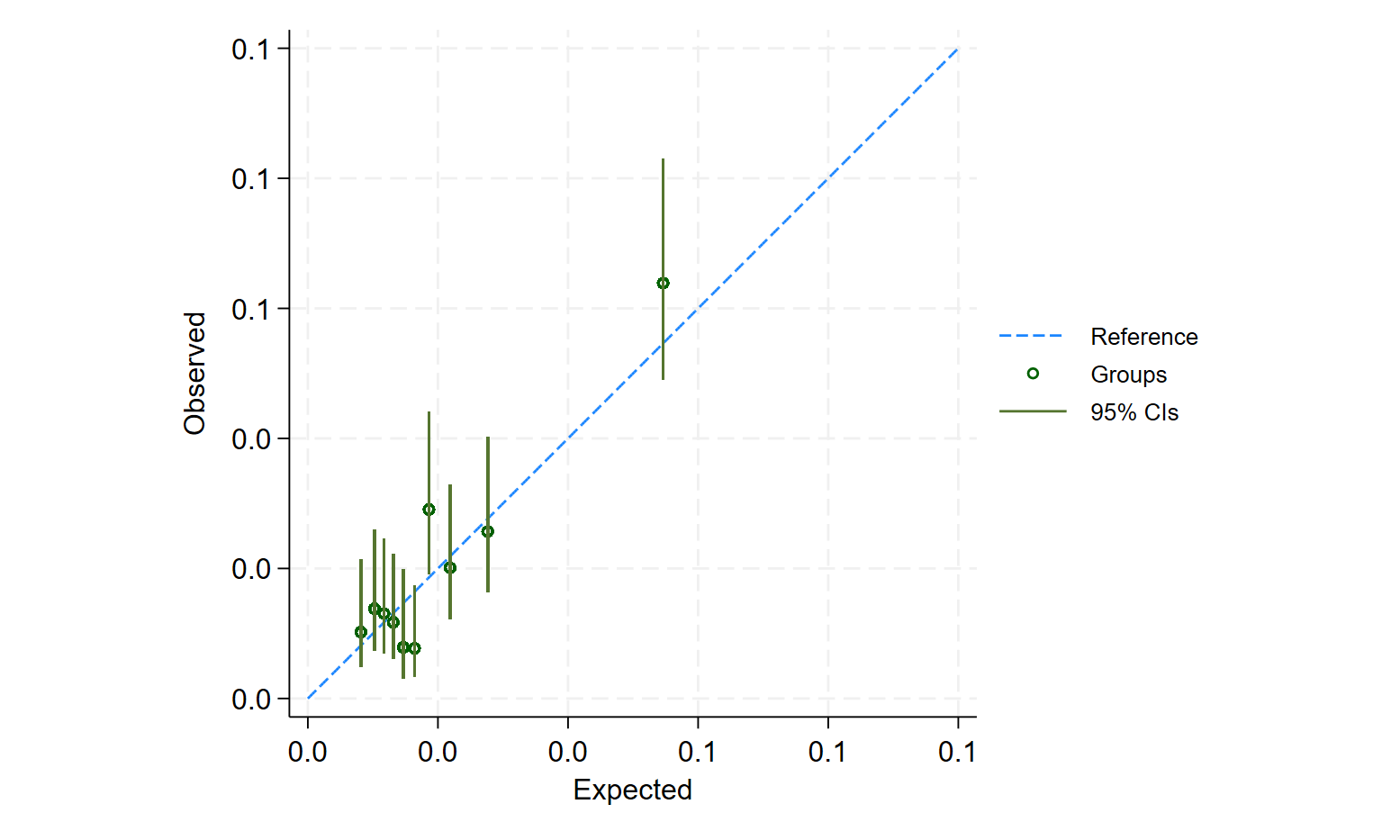


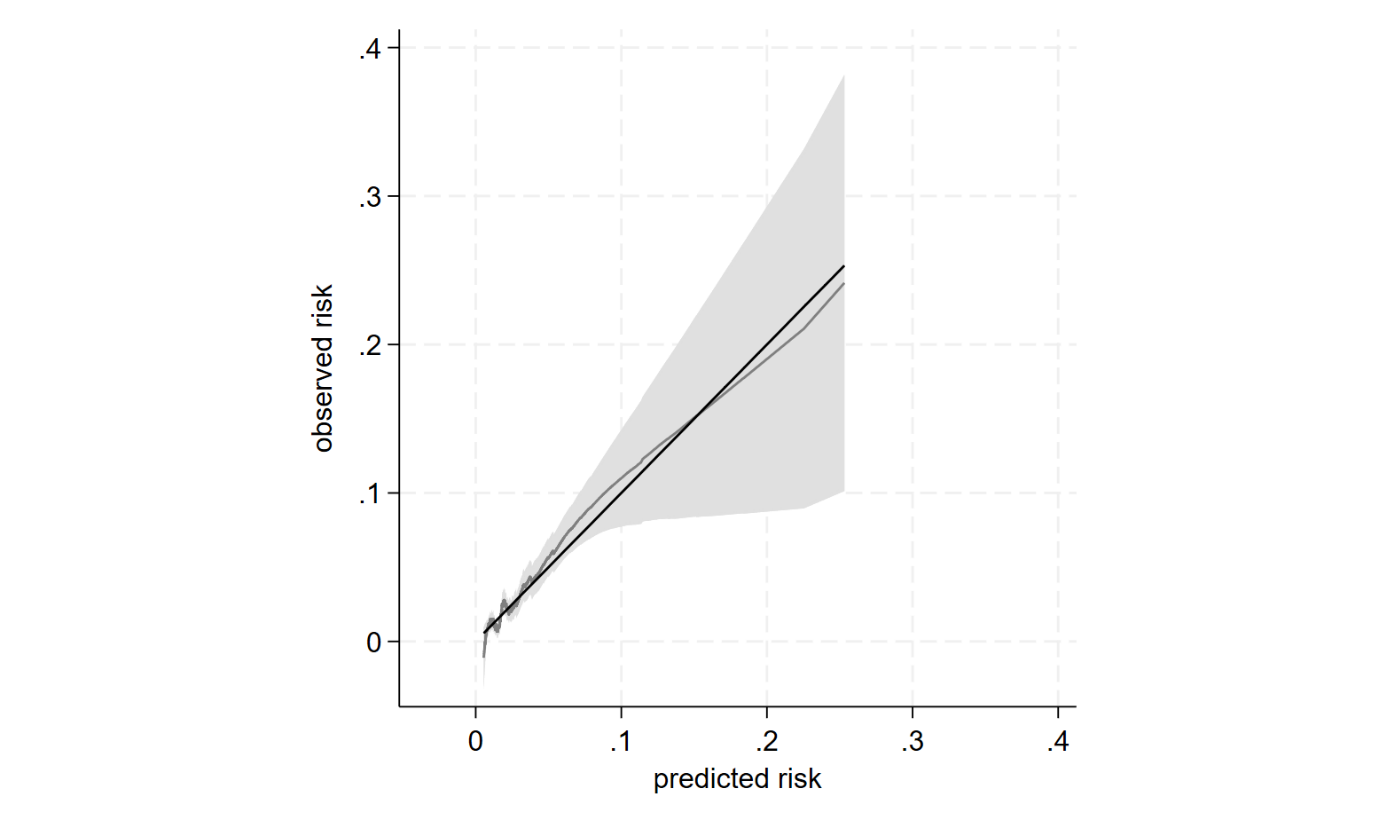


Perfect prediction

Model prediction

95 % CI

Data from a single imputed dataset; So(t=5) =0.994

## Figure S5: Distribution of predicted risk in the model development cohort at 5 years


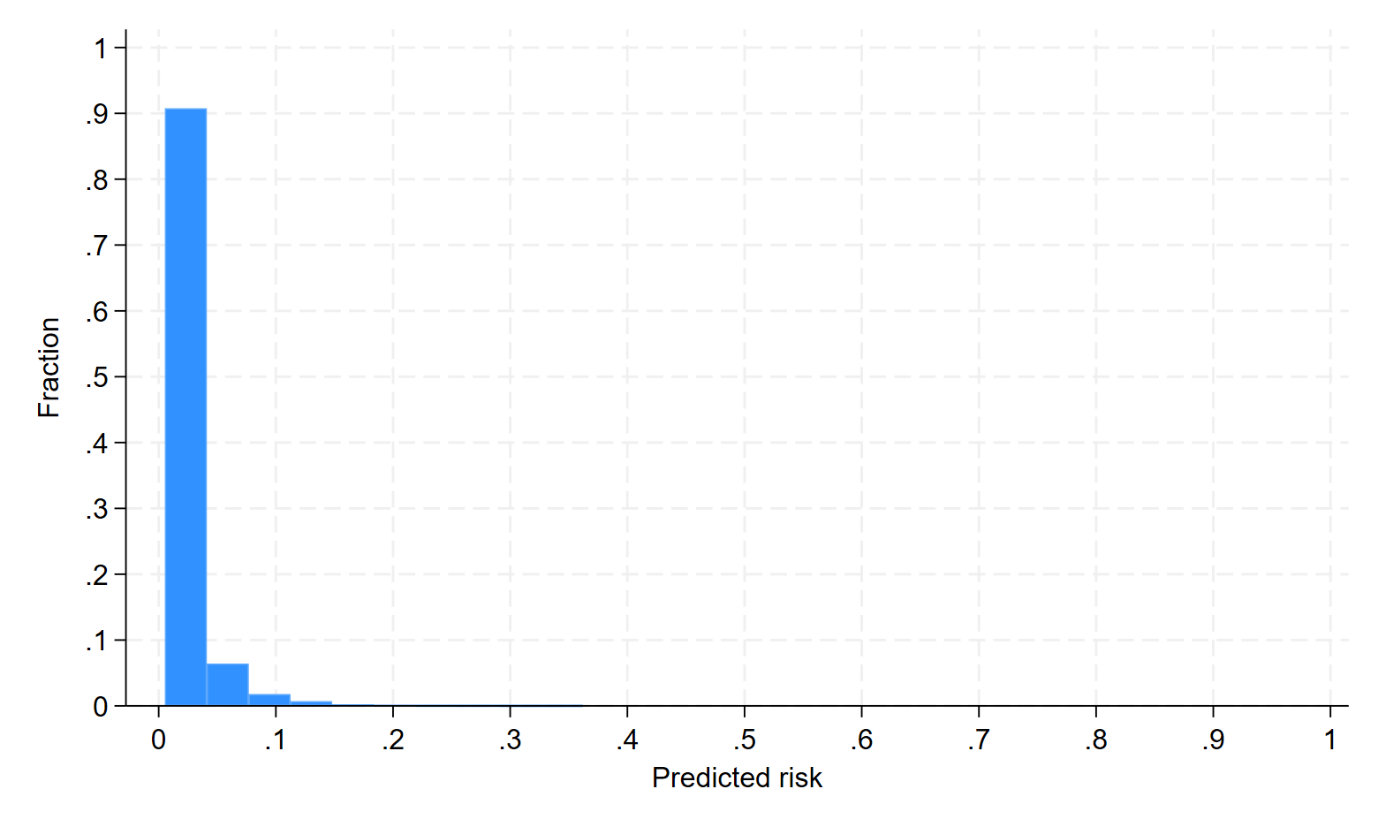


## Figure S6: Calibration plot of a prognostic model for 5-ASA discontinuation with abnormal monitoring blood-test results at 5-years in the validation cohort


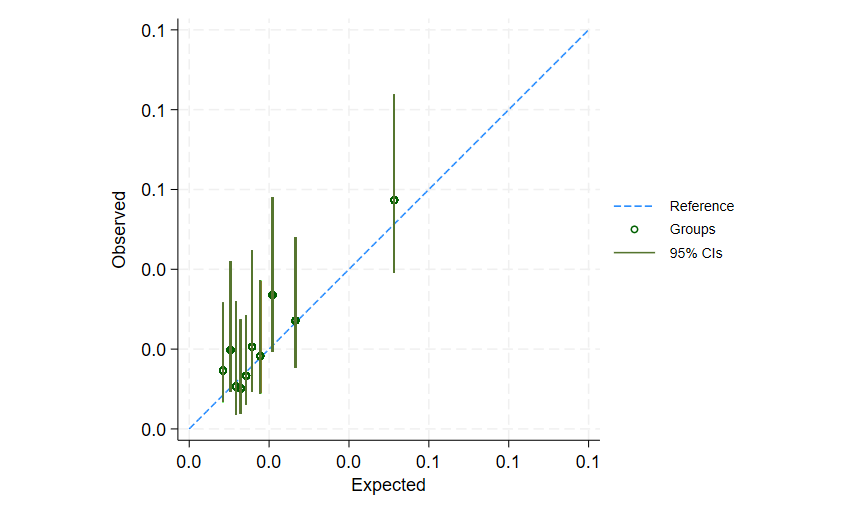


Data from a single imputed dataset; So(t=5) =0.994

## Figure S7: Distribution of predicted risk in the model validation cohort at 5 years


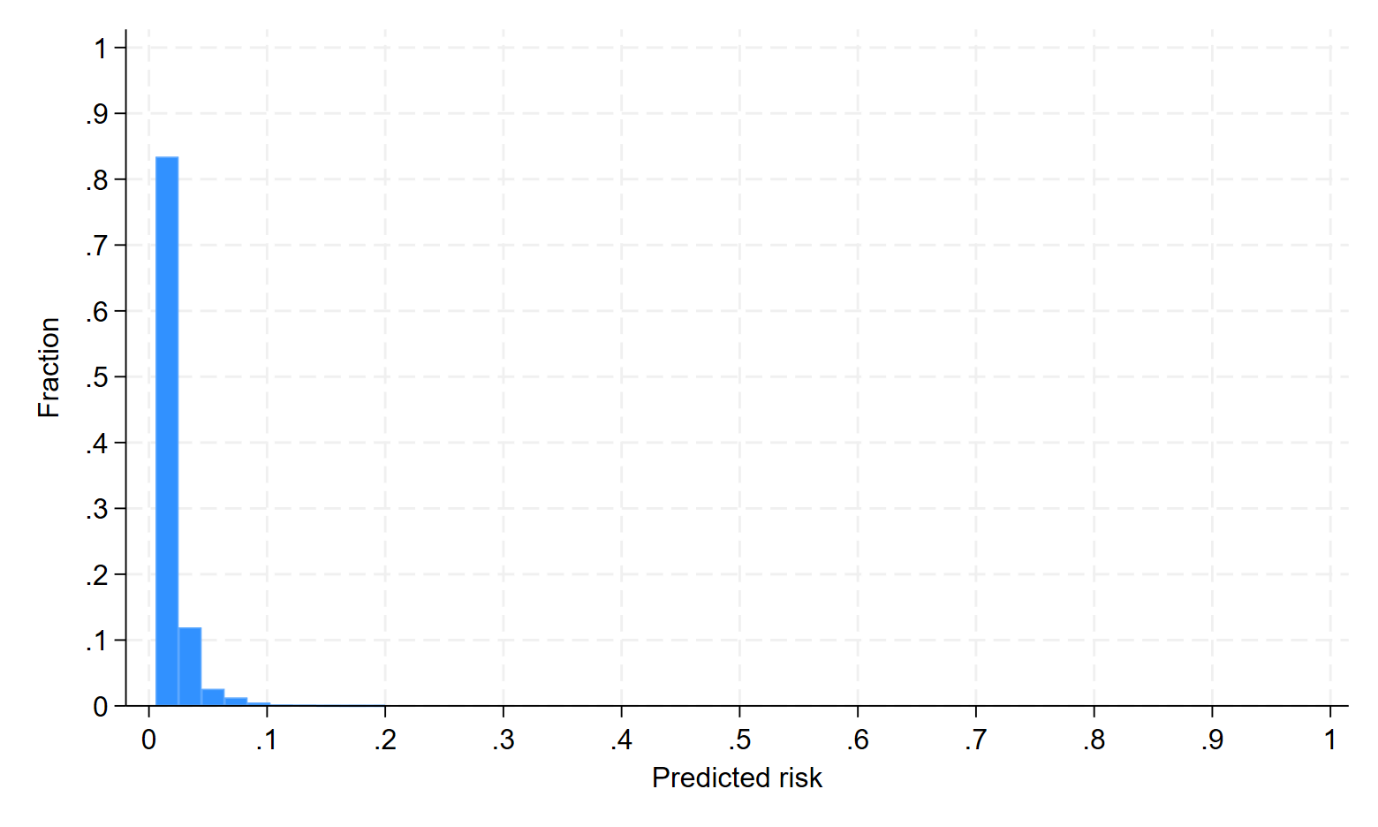


## Figure S8: Calibration of a prognostic model for 5-ASA discontinuation with abnormal monitoring blood-test results at 1 year in the validation cohort


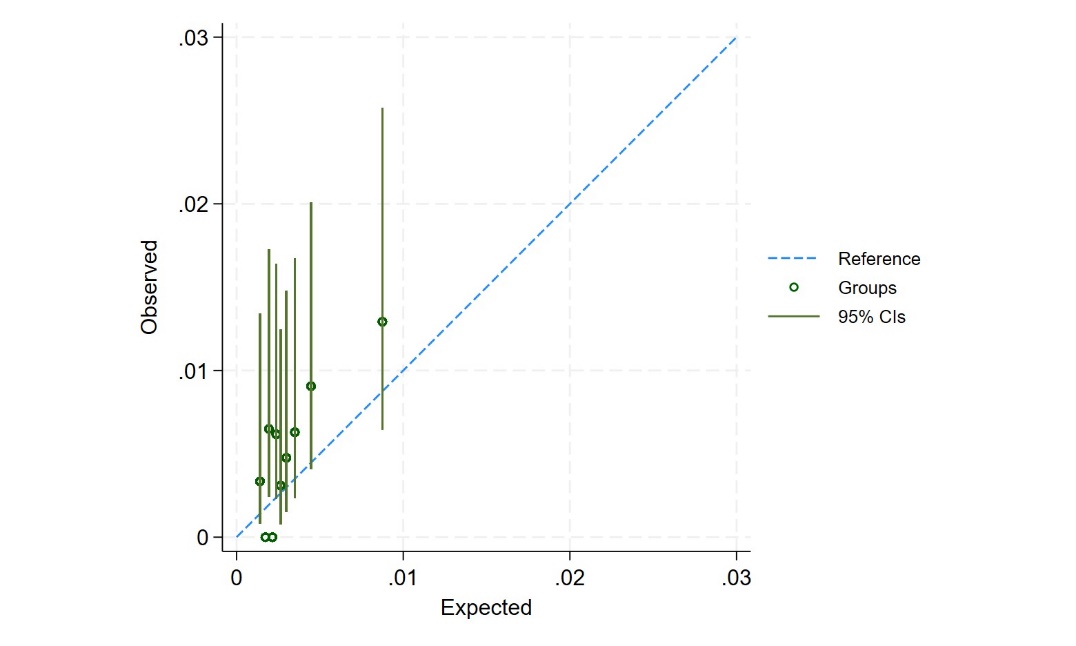


Data from a single imputed dataset was used; s_o_(t_=1_) =0.999

## Figure S9: Calibration of a prognostic model for 5-ASA discontinuation with abnormal monitoring blood-test results at 2 years in the validation cohort

##


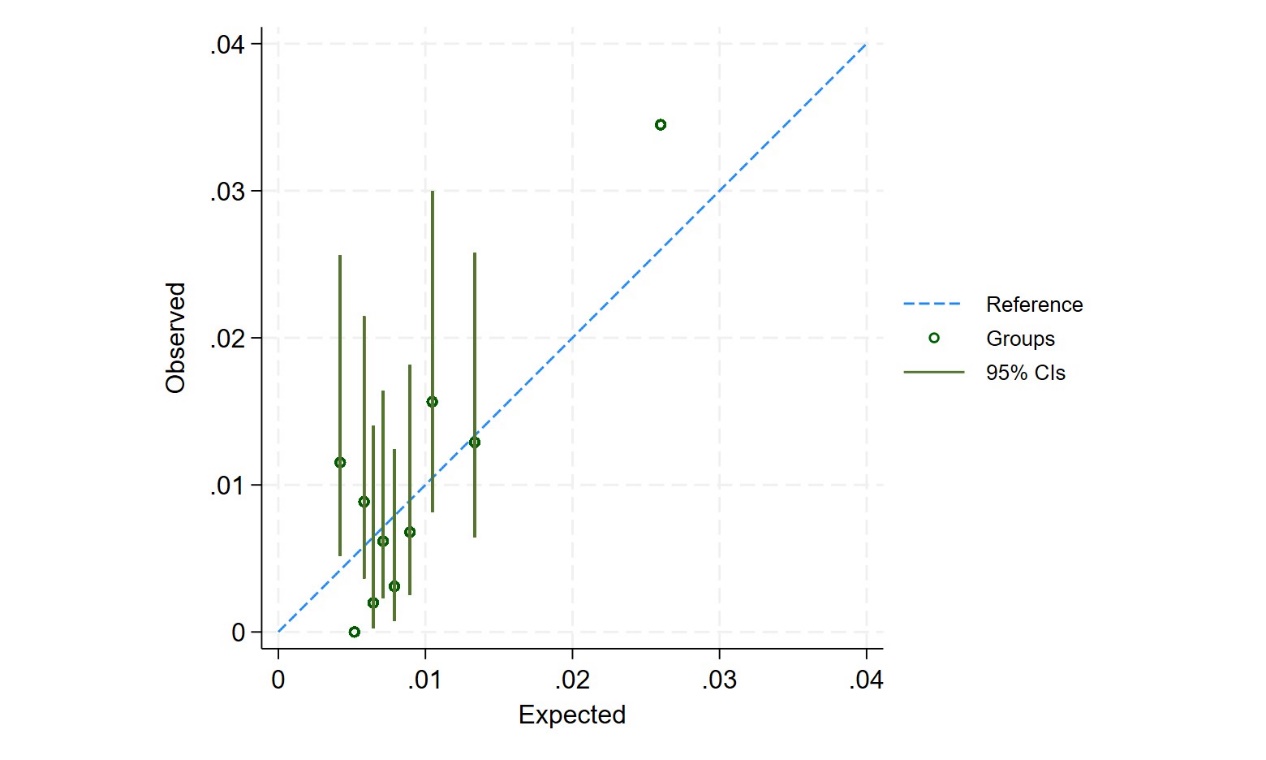


Data from a single imputed dataset was used; s_o_(t_=2_) 0.997

## Figure S10: Calibration of a prognostic model for 5-ASA discontinuation with abnormal monitoring blood-test results at 3 years in the validation cohort


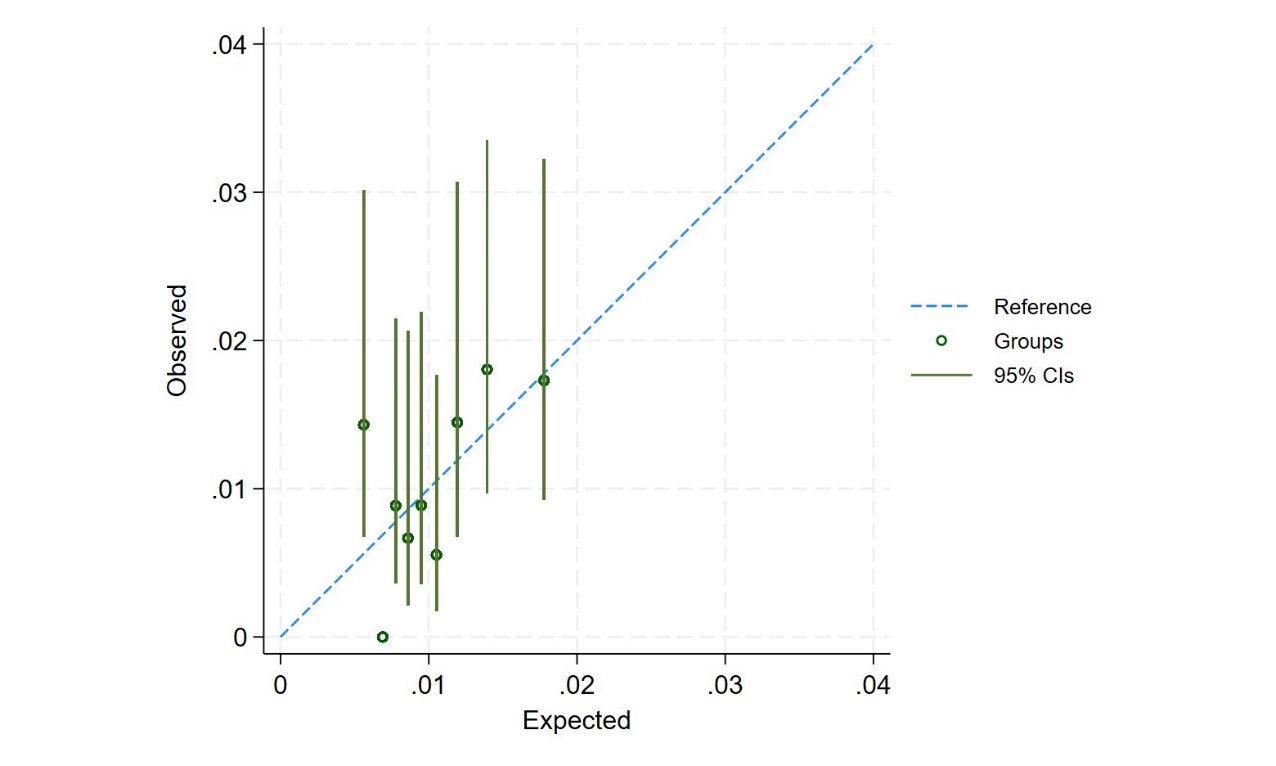


Data from a single imputed dataset was used; s_o_(t_=3_) =0.996

## Figure S11: Calibration of a prognostic model for 5-ASA discontinuation with abnormal monitoring blood-test results at 4 years in the validation cohort


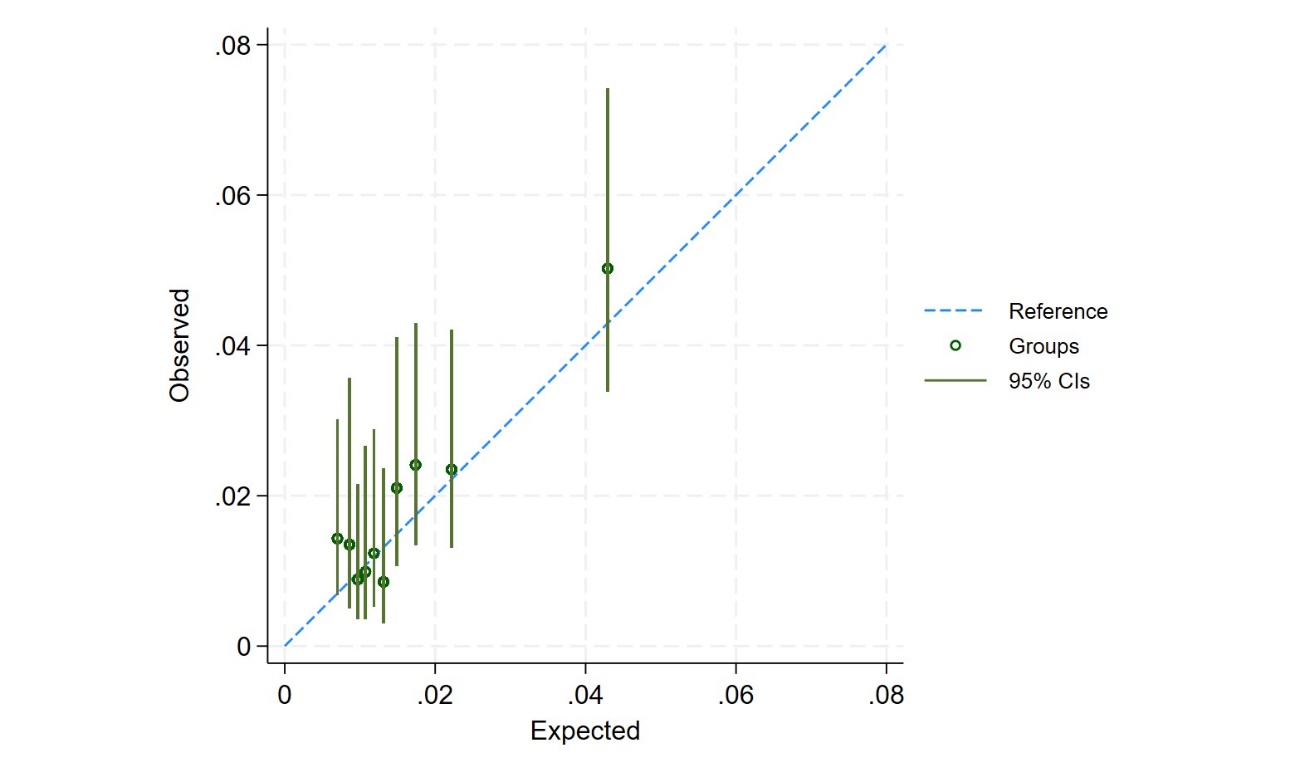


Data from a single imputed dataset was used; s_o_(t_=4_) =0.995
